# Genomic Heritability: What Is It?

**DOI:** 10.1371/journal.pgen.1005048

**Published:** 2015-05-05

**Authors:** Gustavo de los Campos, Daniel Sorensen, Daniel Gianola

**Affiliations:** 1 Departments of Epidemiology & Biostatistics, and Statistics, Michigan State University, United States of America; 2 Department of Molecular Biology and Genetics, Aarhus University, Tjele, Denmark; 3 Departments of Animal Science, Dairy Science and Biostatistics and Medical Informatics, University of Wisconsin-Madison, Madison, Wisconsin, United States of America; Stanford University School of Medicine, United States of America

## Abstract

Whole-genome regression methods are being increasingly used for the analysis and prediction of complex traits and diseases. In human genetics, these methods are commonly used for inferences about genetic parameters, such as the amount of genetic variance among individuals or the proportion of phenotypic variance that can be explained by regression on molecular markers. This is so even though some of the assumptions commonly adopted for data analysis are at odds with important quantitative genetic concepts. In this article we develop theory that leads to a precise definition of parameters arising in high dimensional genomic regressions; we focus on the so-called genomic heritability: the proportion of variance of a trait that can be explained (in the population) by a linear regression on a set of markers. We propose a definition of this parameter that is framed within the classical quantitative genetics theory and show that the genomic heritability and the trait heritability parameters are equal only when all causal variants are typed. Further, we discuss how the genomic variance and genomic heritability, defined as quantitative genetic parameters, relate to parameters of statistical models commonly used for inferences, and indicate potential inferential problems that are assessed further using simulations. When a large proportion of the markers used in the analysis are in LE with QTL the likelihood function can be misspecified. This can induce a sizable finite-sample bias and, possibly, lack of consistency of likelihood (or Bayesian) estimates. This situation can be encountered if the individuals in the sample are distantly related and linkage disequilibrium spans over short regions. This bias does not negate the use of whole-genome regression models as predictive machines; however, our results indicate that caution is needed when using marker-based regressions for inferences about population parameters such as the genomic heritability.

## Author Summary

Whole-genome regression (WGR) methods are being increasingly used for inferring the proportion of variance that can be explained by a linear regression on a massive number of markers, called ‘genomic heritability.’ However, the statistical assumptions involved in WGRs are somewhat at odds with important quantitative genetics concepts. We argue and show that the parameters of the statistical model used for data analysis typically bear a tenuous relationship with the quantitative genetic parameters of interest. We also study, using simulations, the extent of bias of likelihood-based estimates. We conclude that under certain circumstances estimates can have a sizable finite-sample bias; therefore, caution needs to be exercised when interpreting parameter estimates derived from WGR models.

## Introduction

Whole-genome regression (WGR) methods [1] are becoming increasingly used for analysis and prediction of complex traits, quantitative or categorical. These methods were first developed for prediction in plant and animal breeding (e.g., [2,3]). More recently, there has been an increased interest in using WGR methods for inferring the proportion of variance that can be explained by a linear regression on a marker panel, or ‘genomic heritability’ [4–6]. Prediction and inference are two different problems, and a model that may yield good (e.g., unbiased and precise) estimates of parameters of interest may have a relatively poor prediction performance, and vice versa. Most of the methodological research in WGR methods was developed in animal breeding with a focus on prediction. Unfortunately, little is known about the inferential properties of estimates derived from WGRs models. For example, it is unclear whether the commonly used likelihood-based (or Bayesian) estimators of variance components or of genomic heritability estimate population parameters consistently [7].

Before copious marker information became available, genetic analysis (e.g., estimation of heritability) was mainly based on mixed effects linear models applied to family data [8]. In the so-called infinitesimal model, relatedness due to kinship is assessed using pedigrees, and a central element of model specification is the assumption that genotypic values result from the small and additive effects of alleles at a large number of loci. A number of studies have investigated the quality of fit of the infinitesimal model to experimental [9,10] and to simulated family data [11]. Most of these studies have concluded that the additive infinitesimal model is a useful abstraction, except in situations involving a few large-effect non-additive loci. Therefore, at least at some operational level, with family information the distinction between the model that generated the data and the one used for analysis has not seemed critical.

The availability of genotype information on a large number of loci has made possible to assess kinship among nominally unrelated individuals [9–13]. In this setting, and due to imperfect linkage disequilibrium (LD) between markers and quantitative trait loci (QTL), the patterns of allele sharing at markers and at causal loci may be very different [6]. Hence, the distinction between the data generating process and the model used for data analysis, or instrumental model, must be made clearly: in the instrumental model marker genotype information is used in lieu of the causal genotypes that are at the basis of the classical model of quantitative genetic theory. Thus, clarifying the link between the parameters of the instrumental model (e.g., the genomic or SNP variance) and classical quantitative genetic parameters (e.g., the genetic variance) is essential.

Yang et al. (2010) [4] proposed using the G-BLUP method [2], a particular class of WGR, applied to data involving distantly related individuals, for estimation of the proportion of variance accounted for by a multiple-linear regression on common SNP. The proportion of unexplained genetic variance can be interpreted as ‘missing heritability’, which conceptually can be attributed to imperfect LD between markers and QTL. Using a WGR approach Yang et al. (2010) found that approximately half of the heritability of human height was captured by common SNP. Other studies, e.g., [6], have corroborated Yang’s results using both simulated and real data. More recently, WGRs have been used for estimation in scenarios where all causal variants are assumed to be included in the marker panel, and various suggestions have been made with the purpose of obtaining inferences of genomic heritability that resemble more closely those based on pedigrees [5].

In the literature on genomic analysis of complex traits published so far [4–6,14], genetic parameters have been defined based on the statistical assumptions of the instrumental model used for data analysis. This is so despite the fact that there is a key difference in the way genotypes and effects are treated in statistical models and in quantitative genetics theory. In the latter, inter-individual differences in genetic values are attributed to subject-to-subject differences on allele content at QTL [15–17]; therefore genetic variance stems from variation at QTL genotypes. In this framework, at any given time in a population, the effects of alleles on a trait (e.g., the average effects of allele substitution) are fixed quantities, e.g., [16] pp. 112–113. On the other hand, in the instrumental regression models, genotypes are treated as fixed and variation stems from uncertainty about marker effects (the so called ‘variance of marker effects’). This key difference in the treatment of genotypes and of their effects has important consequences that we further explore in this article.

An important contribution of this paper is to establish theory aiming at a precise definition of parameters arising in regression models using genomic data (markers, sequence) as explanatory variables. Our approach is framed within the classical quantitative genetics paradigm. We discuss how these “instrumental model parameters” relate to “structural parameters” of an underlying conceptual QTL model. We also present stylized cases that shed light into the interpretation of the parameters of the instrumental model. Finally, we discuss potential estimation problems and provide a limited set of simulations where some statistical properties of likelihood-based estimates are assessed.

## Materials and Methods

In the first part of this section we develop the basis for a definition of genetic parameters arising in genomic regressions and in the second part of the section we describe two simulation studies conducted to assess some statistical properties of likelihood-based estimates of genomic parameters.

### Theory

In standard quantitative genetic theory [15–17] additive genetic values are linear functions of allele content at QTL. Concepts such as the additive effect of an allele in a population or narrow sense (trait) heritability are defined with reference to this framework. The standard quantitative genetic model (hereinafter referred to as ***QTL-model*)** assumes that a trait of interest measured on individual *i* (*y*_*i*_; *i* = 1, …, n) is affected by alleles at *q* QTL. Hereinafter, for ease of presentation, we assume that all loci are bi-allelic and that genotype codes (*z*_*ij*_; *j* = 1,2, …, q) and phenotypes have been centered, so that *E*(*y*_*i*_) = 0 and *E(z*_*ij*_) = 0 for all individuals and loci.

The ***genetic value*** of an individual is defined as the expected phenotypic value given QTL genotypes, *g*_*i*_ = *E*(*y*_*i*_|*z*_*i*_), where *z*_*i*_ = {z_*i*1, …,_ z_*iq*_} is a vector of genotype codes observed at the *i* ^*th*^ individual at each of the *q* QTL. The conditional expectation function maps from genotypes to expected phenotypic value. The **genetic variance** of a trait in the conceptual population is simply the average (over individuals) squared deviation of the genetic values from the population mean. In our setting, because *E*(*y*_*i*_) = 0, the genetic variance becomes $\sigma_{g}^{2}\;=\;E_{z}\left\{{E_{y|z\;}\left(y_{i}|z_{i}\right)}^{2}\right\}\;=\;E_{z}\left\{g_{i}^{2}\right\}$. Clearly, genetic variation stems from inter-individual differences in allele content at QTL (this is what confers individuals different genetic values).

#### Additive effects and additive variance

The conditional expectation function, *ɡ*_*i*_ = *E*(*y*_*i*_ | *z*_*i*_), may not be linear on QTL effects. However, regardless of the genetic mechanism operating within or across loci, one can always define a linear regression model of the form

$$y_{i}\;=\;\alpha 'z_{i}+\delta_{i},$$ (1)

where *α*, a column vector of dimension *q*, whose elements are the regressions of phenotype on allelic content, represents the vector of ***additive effects of the QTL***, or effects of allele substitutions [16], defined as the regression of *ɡ*_*i*_ on *z*_*i*_. Thus

α = Covzi,zi' -1Covzi,gi = Σz-1Σzg. (2)

Here,

$${\;\Sigma }_{z}\;=\;\left[\begin{array}{ccc} Var(z_{i1}) & \ldots & Cov(z_{i1},z_{iq})\\ \vdots & \ddots & \vdots \\ Cov(z_{iq},z_{i1}) & \ldots & Var(z_{iq}) \end{array}\right]$$

is a *q*×*q* matrix whose entries are the variances and covariances of allelic contents at the *q* QTL, and $\Sigma_{zg}^{}\;=\;{\left\{Cov\left(z_{i1},g_{i}\right),\ldots ,Cov\left(z_{iq},g_{i}\right)\right\}}^{'}$ is a q-dimensional vector containing covariances between genotypes at QTL and genetic values. In (1), the deviate *δ*_*i*_ is a random residual that includes genetic and environmental effects that cannot be captured by the linear regression on allele contents, e.g., dominance, epistasis and QTL-environment interactions. By construction this residual is uncorrelated with QTL genotypes, indeed

Covyi-α'zi,zi' = Covyi,zi'-Covα'zi,zi' = Covgi,zi'-ΣgzΣz-1Σz = Σgz-Σgz = 0

Importantly, the terms $\Sigma_{z}^{}$ and $\Sigma_{zg}^{},\;$ and therefore *α*, are viewed as fixed population quantities and not as random variables. Also, these covariance matrices are assumed to be homogeneous across individuals; this condition may be met in an unstructured population but may not hold if individuals within the population cluster according to some substructure or are aggregated in families because, in such cases, variances and covariances may vary among clusters. On the other hand, α′z_*i*,_ is random because QTL genotypes vary between individuals in the population according to some law such as Hardy-Weinberg.

(Eq 1) leads to the following decomposition of phenotypic variance

$$Var\left(y_{i}\right)\;=\;Var\left(\alpha 'z_{i}\right)+Var\left(\delta_{i}\right)$$

or

$$\sigma_{y}^{2}\;=\;\alpha '\Sigma_{z}\alpha +\sigma_{\delta }^{2}\;=\;\sigma_{a}^{2}+\sigma_{\delta }^{2},$$

where

$$\sigma_{a}^{2}\;=\;\sum_{j\;=\;1}^{q}Var\left(z_{ij}\right)\alpha_{j}^{2}+2\sum_{j\;=\;1}^{q}\sum_{j^{'}>j}^{q}Cov\left(z_{ij},z_{ij^{'}}\right)\alpha_{j}\alpha_{j^{'}}$$ (3)

is the ***additive genetic variance***, stemming from the regression of phenotype on allelic contents at QTL. Note that in (3) randomness arises from variation and covariation of allelic contents at the QTL, as postulated in the basic model of quantitative genetics [15–17],whereas *α* is a fixed parameter.

Expression (3) shows that the additive variance is not only a function of the variances of QTL genotypes (the diagonal elements of *Σ*_*z*_) and of their effects, but also of the patterns of LD between QTL (the off-diagonal elements of *Σ*_*z*_). The contribution of LD to genetic variance (additive and non-additive) is a well-established result in quantitative genetics [18,19]. For this reason, in general, the additive variance cannot be partitioned into locus-specific components [19,20]. For example, following Avery and Hill [19] if in a large population individuals mate completely at random, the additive variance can be expressed as

$$\sigma_{a}^{2}\;=\;{2\Sigma }_{j\;=\;1}^{q}\theta_{j}\left(1-\theta_{j}\right)\alpha_{j}^{2}+4\Sigma_{j\;=\;1}^{q}\Sigma_{j^{'}>j}^{q}\alpha_{j}\alpha_{j'}D_{jj'}^{}$$

where $D_{jj'}^{}$ is the coefficient of LD between locus *j* and *j’* and *θ*_*j*_ is the frequency of the allele coded as 1 at the *j* ^*th*^ QTL [19]. However, if alleles at QTL pairs are in complete LE, $D_{jj'}^{}\;=\;0\;for\;j\neq j^{'}$, leading to $\sigma_{a}^{2}\;=\;\sum_{j\;=\;1}^{q}2\theta_{j}\left(1-\theta_{j}\right)\alpha_{j}^{2}$. In this case, the additive variances at each of the QTL “add up” to the total additive genetic variance.

***Narrow sense (trait) heritability*** is defined as the proportion of phenotypic variance explained by additive effects [15–17], that is

$$h^{2}\;=\;\frac{\sigma_{a}^{2}}{\sigma_{y}^{2}}\;=\;\frac{\sigma_{a}^{2}}{\sigma_{a}^{2}+\sigma_{\delta }^{2}}\;=\;\frac{\alpha '\sum_{z}\alpha }{\alpha '\sum_{z}\alpha +\sigma_{\delta }^{2}}$$

Note that *h* ^2^ is also the squared correlation between phenotypes and additive genetic values, since

$$Cor{\left(y_{i,},\alpha 'z_{i}\right)}^{2}\;=\;\frac{Cov{\left(y_{i,},\alpha 'z_{i}\right)}^{2}}{Var\left(y_{i}\right)Var(\alpha {'z}_{i})}\;=\;\frac{Var{\left(\alpha 'z_{i}\right)}^{2}}{Var\left(y_{i}\right)Var(\alpha {'z}_{i})}\;=\;h^{2}.$$

Therefore, *h* ^2^ is also the maximum r-squared that can be achieved when fitting a linear additive model on known QTL allelic contents.

#### Instrumental Model (regression on markers)

While genetic values are functions of allele content at QTL, in practice the set of genes affecting a complex trait is often unknown. Therefore empirical (instrumental) linear regression models are fitted using markers whose alleles are typically in imperfect LD with those at QTL. Concepts such as the “additive effect of a marker”, or the amount of variance explained by marker effects (the so called “genomic variance”) have been employed by many authors (e.g., Goddard 2009). However, the literature lacks a precise definition of these parameters as well as of an analytical treatment that holds regardless of trait architecture or patterns of LD. Here, we attempt to fill this gap by presenting precise definitions of marker effects and of genomic heritability, from a quantitative genetic theory perspective.

Suppose that the analysis is carried out using *p* markers with genotype codes in vector *x*_*i*_ = {*x*_*i*1_, …, *x*_*ip*_} ′; as before, we assume that marker genotypes have been centered, that is *E*{*x*_*ij*_} = 0 (*j* = 1,...,*p*). The marked additive genetic value can be defined as the regression of the true additive genetic value, *α′*z_*i*_, on allelic content at marker loci, that is

$$\alpha 'z_{i}\;=\;{\beta 'x}_{i}+\xi_{i},$$ (4)

where ξ_*i*_ is a model residual representing components of the true additive genetic values that, due to imperfect marker-QTL LD, cannot be explained by a regression on markers.

***Marker effects*** are defined as the multivariate multiple regression of additive genetic values on markers

$$\beta \;=\;Var{\left(x_{i}\right)}^{-1}Cov\left(x_{i},\alpha 'z_{i}\right)\;=\;{\Sigma_{x}^{-1}\Sigma }_{xz}\alpha \;=\;B\alpha$$ (5)

where,

$$\Sigma_{x}^{}\;=\;\left[\begin{array}{ccc} Var(x_{i1}) & \ldots & Cov(x_{i1},x_{ip})\\ \vdots & \ddots & \vdots \\ Cov(x_{ip},x_{i1}) & \ldots & Var(x_{ip}) \end{array}\right],$$

is the *p×p* covariance matrix among marker genotypes,

$$\Sigma_{xz}\;=\;\left[\begin{array}{ccc} Cov(x_{i1},z_{i1}) & \ldots & Cov(x_{i1},z_{iq})\\ \vdots & \ddots & \vdots \\ Cov(x_{ip},z_{i1}) & \ldots & Cov(x_{ip},z_{iq}) \end{array}\right]$$

is a *p×q* matrix of covariances between marker and QTL genotypes, and $B\;=\;{\Sigma_{x}^{-1}\Sigma }_{xz}\;=\;\left\{b_{x_{j}z_{k}}\right\}$ is *p×q* a matrix containing regressions on allelic contents of each QTL on each marker. Since $\Sigma_{x}^{}$, $\Sigma_{xz}^{}$ and *α* are fixed population parameters, so is *β*. Also, note that, by definition, the regression residual ξ_i_ = z_i_ ′*α—x*_*i*_ *′β* is uncorrelated with *x*_*i*_. Indeed

Covxi,ξi = Cov(xi,zi'α-xi'β) = Σxzα- Σxβ = Σxzα- ΣxΣx-1Σxzα = 0.

***Genomic values*** are then defined as $\beta 'x_{i}\;=\;\alpha^{'}\Sigma_{zx}\Sigma_{x}^{-1}x_{i}\;=\;\alpha '\;{\hat{z}}_{i}$, where ${\hat{z}}_{i}\;=\;\Sigma_{zx}\Sigma_{x}^{-1}x_{i}\;=\;B'x_{i}$ is the best linear predictor of allele content at QTL, given allele content at markers. Note that while each element of *z*_*i*_ takes one of 3 exhaustive and mutually exclusive values, ${\hat{z}}_{i}$ is a continuous-valued vector, this being due to the linear approximation used. The variance of genomic values, or ***genomic variance,*** is

$$\begin{array}{l} Var\left(\beta \prime x_{i}\right)=\beta \prime Cov\left(x_{i},x_{i}\prime \right)\beta \\ \;=\beta \prime \Sigma_{x}^{}\beta \\ \;=\alpha \prime \Sigma_{zx}\Sigma_{x}^{-1}\Sigma_{x}^{}\Sigma_{x}^{-1}\Sigma_{xz}\alpha \\ \;=\alpha \prime \Sigma_{zx}\Sigma_{x}^{-1}\Sigma_{xz}\alpha \; \end{array}$$ (6)

Its value depends on the QTL effects (*α*), and on the LD relationships among QTL and markers (via *Σ*_*xz*_), and among markers (via $\Sigma_{x}^{-1}$). The variance of the regression residual is

$${Var(\xi }_{i})\;=\;Var\left(\alpha 'z_{i}-{\beta 'x}_{i}\right)\;=\;\alpha '\;\Sigma_{z}\alpha -\alpha '\Sigma_{zx}\Sigma_{x}^{-1}\Sigma_{xz}\alpha \;=\;\alpha '\;(\Sigma_{z}-\Sigma_{zx}\Sigma_{x}^{-1}\Sigma_{xz})\alpha$$

The expression between parentheses in the right-hand side is the conditional (if the joint distribution were multivariate normal) covariance matrix among QTL genotypes, given markers. This quantity represents remaining uncertainty about QTL genotypes once markers are observed. Because ξ_*i*_ is uncorrelated with *x*_*i*_, the model in (Eq 4) yields the variance partition

$$Var\left(\alpha 'z_{i}\right)\;=\;Var\left({\beta 'x}_{i}\right)+Var\left(\xi_{i}\right),$$

leading to the decomposition

$$\alpha '\Sigma_{z}\alpha \;=\;\alpha '\Sigma_{zx}\Sigma_{x}^{-1}\Sigma_{xz}\alpha \;+\alpha '(\Sigma_{z}-\Sigma_{zx}\Sigma_{x}^{-1}\Sigma_{xz})\alpha$$

or

$$\sigma_{a}^{2}\;=\;\sigma_{g}^{2}+\sigma_{\overset{-}{g}}^{2}$$

where, $\sigma_{g}^{2}\;=\;Var\left({\beta 'x}_{i}|\alpha \right)$ is the variance of the ‘genomic values’ or genomic variance, see expression (6), is interpretable as the amount of additive variance captured by regression on markers. Likewise, $\sigma_{\overset{-}{g}}^{2}$ can be interpreted as the “missing” additive genetic variance, that is, the variability yet to be marked. The ratio $\sigma_{g}^{2}/\sigma_{a}^{2}$ represents the proportion of additive variance that is explained by a linear regression on available markers and the product of this ratio times the trait heritability is the proportion of variance of phenotypes explained by the regression on markers, or ***genomic heritability***:

$$h_{g}^{2}\;=\;\frac{\sigma_{g}^{2}}{\sigma_{y}^{2}}\;=\;\frac{\sigma_{a}^{2}}{\sigma_{y}^{2}}\frac{\sigma_{g}^{2}}{\sigma_{a}^{2}}\;=\;h^{2}\frac{\sigma_{g}^{2}}{\sigma_{a}^{2}}$$

The proportion of ***missing heritability*** can be defined as a population parameter as

$$\frac{h^{2}-h_{g}^{2}}{h^{2}}\;=\;\frac{{\sigma_{a}^{2}-\sigma }_{g}^{2}}{\sigma_{a}^{2}}\;=\;\frac{\alpha^{'}\Sigma_{z}\alpha -\alpha '\Sigma_{zx}\Sigma_{x}^{-1}\Sigma_{xz}\alpha }{\alpha^{'}\Sigma_{z}\alpha }\;=\;\frac{\alpha '{(\Sigma_{z}-\Sigma }_{zx}\Sigma_{x}^{-1}\Sigma_{xz})\alpha }{\alpha '\Sigma_{z}\alpha }$$

The covariance between phenotypes and genomic values is *Cov*(*y*_*i*_,*β*′*x*_*i*_) = *Cov*(*β*′*x*_*i*_ + *ξ*_i_ + *δ*_i_,β′*x*_*i*_) = *Var*(*β*′*x*_*i*_) + *Cov*(*ξ*_i,_ *β*′*x*_*i*_) + *Cov*(*δ*_i_,*β*′*x*_*i*_), The 1^st^ term on the right-hand-side of the preceding equation is the genomic variance and the second term is, by construction, zero. However, the term *Cov*(*δ*_i_,*β*′*x*_*i*_) may not be zero because *δ*_*i*_ and *x*_*i*_ may be correlated. However, if *Cov*(*δ*_i_,*β*′*x*_*i*_) = 0, then $Cov\left(y_{i},\beta 'x_{i}\right)\;=\;Var\left(\beta 'x_{i}\right)\;=\;\alpha '\Sigma_{zx}\Sigma_{x}^{-1}\Sigma_{xz}\alpha$, and the squared-correlation between phenotypes and genomic values becomes, $Cor{\left(y_{i},\beta 'x_{i}\right)}^{2}\;=\;\frac{\left(\alpha '\Sigma_{zx}\Sigma_{x}^{-1}\Sigma_{xz}\alpha \right)}{\sigma_{y}^{2}}\;=\;h_{g}^{2}$.

#### Rotation invariance

The additive and genomic variances (expressions 3 and 6, respectively) are invariant under linear transformations of genotypes; therefore, these parameters, and functions thereof, do not depend on how genotypes are coded. A proof of this property is provided in the Supplementary methods S1 Text.

#### Insights from special cases

Above, parameters of the instrumental model were defined without imposing any restriction on patterns of LD. Special cases that shed light into interpretation of some of these parameters can be obtained by imposing restrictions on the trait ‘architecture’ and on the patterns of LD.

For instance, if there is a ***single marker-QTL pair***, both *Σ*_*zx*_ and *Σ*_*x*_ are scalars; therefore, the marker effect (population parameter), using (Eq 5) becomes

$$\beta \;=\;{\Sigma_{x}^{-1}\Sigma }_{xz}\alpha \;=\;b_{zx}\alpha$$

where $b_{zx}\;=\;\frac{cov{\left(x_{i},z_{i}\right)}^{}}{var\left(x_{i}\right)}$ is the linear regression of the QTL genotype on the marker genotype. The genomic value is then *x*_*i*_ *β = x*_*i*_ *b*_*zx*_ *α*, and the expression for the genomic variance (Eq 6) reduces to $\sigma_{g}^{2}\;=\;\frac{cov{\left(x_{i},z_{i}\right)}^{2}}{var\left(x_{i}\right)}\alpha^{2}$. The proportion of the additive variance explained by the regression on markers is

$$\frac{\sigma_{g}^{2}}{\sigma_{a}^{2}}\;=\;\frac{\left[\frac{cov{\left(x_{i},z_{i}\right)}^{2}}{var\left(x_{i}\right)}\alpha^{2}\right]}{\left[{var\left(z_{i}\right)\alpha }^{2}\right]}\;=\;\frac{cov{\left(x_{i},z_{i}\right)}^{2}}{var\left(z_{i}\right)var\left(x_{i}\right)}\;=\;r^{2},$$

the squared correlation between genotypes at the marker locus and at the QTL. Therefore, the genomic heritability is $h_{g}^{2}\;=\;r^{2}h^{2}\leq h^{2}$. If LD is perfect, $h_{g}^{2}\;=\;h^{2}$; otherwise, it will get closer to 0 as LD becomes weaker.

Goddard (2009)[21] used a conceptual framework where QTL are in mutual LE and, for each QTL, there is a single marker associated to it. In this stylized setting the entire genome is represented as ***independent QTL-markers pairs*** (*z*_ij_, *x*_*ij*_). Under these conditions several simplifications occur. Because of LE between markers and QTL in different pairs, the matrices *Σ*_*x*_ and *Σ*_*z*_ are diagonal. Further, if markers and QTL are sorted by pair, then *Σ*_*zx*_ is also diagonal. In this setting marker effects are simply obtained by regressing QTL genotypes on markers within pairs, that is, $\beta_{j}\;=\;b_{z_{j}x_{j}}\alpha_{j}$ where $b_{z_{j}x_{j}}\;=\;\frac{Cov\left(x_{ij},z_{ij}\right)}{Var\left(x_{ij}\right)}$ is the regression of the *j* ^*th*^ QTL on the *j* ^*th*^ marker. The genomic variance can be decomposed as the sum of marker-specific components:

$$\sigma_{g}^{2}\;=\;\sum_{j}^{}\frac{cov{\left(x_{ij},z_{ij}\right)}^{2}}{var\left(x_{ij}\right)}\alpha_{j}^{2}\;=\;\sum_{j}var\left(x_{ij}\right)\beta_{j}^{2}\;=\;\sum_{j}var\left(z_{ij}\right)r_{j}^{2}\alpha_{j}^{2}$$ (7)

where $r_{j}^{2}$ is the squared-correlation between the marker and the QTL genotype at the *j* ^*th*^ pair (Goddard, 2009).

With ***sequence data*** all “causal variants” are expected to be included in the marker panel; therefore, it is reasonable to expect that there will be no missing heritability, i.e., the trait heritability and genomic heritability parameters coincide. The framework outlined in previous sections is consistent with this view. In fact, it can be shown that when all “causal variants” are included in the marker panel, the vector of marker effects satisfies:*β*_*j*_ = {α_j_ *if x*_*j*_ is *a QTL*; 0 *otherwise*}. Consequently, the genomic and trait heritability coincide (i.e., there is no missing heritability). This intuitive but important result can be derived using properties of inverses of partitioned matrices, and a detailed derivation is provided in the Supplementary Methods S2 Text. Importantly, this result applies to the relationship between the trait heritability and genomic heritability parameters as defined in expressions 3 and 7. However, this does not imply that estimators would converge in probability to the true population parameters; we return to this problem in the section on parameter estimation.

#### On defining genomic parameters based on statistical assumptions

As stated earlier, in classical quantitative genetics theory genetic variance arises from variation and co-variation of allelic contents at QTL, and both QTL and marker effects are fixed population parameters. However, in WGR studies marker genotypes are observable but their effects are unknown. In fact, in classical likelihood or Bayesian analysis, marker effects are treated as random variables, mainly because the number of markers exceeds samples size and restrictions must be imposed on the values of the regressions, and inferences are conditional on the observed marker genotypes. For example, in the family of models named the Bayesian alphabet [22,23], marker effects $b\;=\;{\left\{b_{j}\right\}}_{j\;=\;1}^{p}$ are assumed to be IID (identically and independently distributed) draws from some common prior distribution with null mean and variance ${Var(b}_{j})\;=\;\sigma_{b}^{2}$. The regression model is built conditionally on the observed marker genotypes and the prior variance of the *i* ^*th*^ genomic value is: $Var\left(b{'x}_{i}|x_{i}\right)\;=\;\sigma_{b}^{2}\sum_{j}x_{ij}^{2}$. The expected value of this parameter taken over the distribution of marker genotypes is ${\sigma_{u}^{2}\;=\;E}_{x_{i}}\left\{Var\left(b{'x}_{i}|x_{i}\right)\right\}\;=\;\sigma_{b}^{2}\sum_{j}E\left(x_{ij}^{2}\right)\;=\;\sigma_{b}^{2}\sum_{j}Var\left(x_{ij}^{}\right)$. Under HW-equilibrium, $Var\left(x_{ij}^{}\right)\;=\;2\pi_{j}\left(1-\pi_{j}\right),$ where π_*j*_ is the allelic frequency at marker locus *j*. Therefore

$$\sigma_{u}^{2}\;=\;E_{x_{i}}\left\{Var\left(b{'x}_{i}|x_{i}\right)\right\}\;=\;\sigma_{b}^{2}\sum_{j}2\pi_{j}\left(1-\pi_{j}\right)$$ (8a)

If the $x_{ij}^{}$ are standardized to have unit variance,

$$\sigma_{u}^{2}\;=\;p\sigma_{b}^{2}$$ (8b)

In the literature, expressions (8a) and (8b) are usually referred to as the “genomic variance” (e.g., VanRaden 2008). However, for the reasons discussed above, the link between expression (8a) and the population parameter $\sigma_{g}^{2}$ defined in (6) is not straightforward. First, as stated, from a quantitative genetics perspective, marker effects are fixed population parameters and not random variables. Secondly, expression (8a) suggests that the genomic variance can be decomposed into locus-specific components. However as noted earlier, under general conditions this is not possible, because LD affects both the additive and the genomic variances, precluding a decomposition such as that implied by the right hand side of expressions (8a) and (8b).

To illustrate, consider a simplified setting with one QTL and two markers, and assume that genotypes are standardized to unit variance and that *α* = 1, $\Sigma_{x}\;=\;\left[\begin{array}{cc} 1 & .5\\ .5 & 1 \end{array}\right]$, and *Σ*_*zx*_ = [.5 .5]. Using expression (5) in this setting produces marker effects that are both equal to 1/3. If we ignore the LD between markers, the individual contribution to genomic variance of each marker locus is (1/3)^2^, leading to a total genomic variance equal to 2/9. However, using (6) we obtain that the genomic variance is considerably larger $\left[\begin{array}{cc} .5 & .5 \end{array}\right]{\left[\begin{array}{cc} 1 & .5\\ .5 & 1 \end{array}\right]}^{-1}\left[\begin{array}{c} .5\\ .5 \end{array}\right]\;=\;\frac{1}{3}$. In order to express the genomic variance in a form that is similar to the genomic variance parameter of the instrumental model (8a), it is necessary to assume the stylized model that leads to expression (7).

This shows the rather tenuous relationship between the population parameter $\sigma_{g}^{2}$ and the parameter of the statistical model $\sigma_{u}^{2}$.

Above we demonstrated the consequences of ignoring LD in the determination of the genomic variance. If markers are in complete mutual linkage equilibrium expression (6) reduces to $\sigma_{g}^{2}\;=\;\beta^{'}\Sigma_{x}^{}\beta \;=\;\sum_{j}2\pi_{j}\left(1-\pi_{j}\right)\beta_{j}^{2}$; this is equivalent to the statistical parameter $\sigma_{u}^{2}\;=\;\sigma_{b}^{2}\sum_{j}2\pi_{j}\left(1-\pi_{j}\right)$ only if all effects have equal size, that is *β*_1_ = *β*_*2*_ = … = *β*_*0*_, with $\beta_{0}^{2}\;=\;\sigma_{b}^{2}$. If effects have unequal size and there is no association between effect size and allele frequency, then one may regard $\sigma_{b}^{2}\sum_{j}2\pi_{j}\left(1-\pi_{j}\right)$ as a reasonable approximation to $\sigma_{g}^{2}$ by interpreting $\sigma_{b}^{2}$ as the ‘average squared-effect’. However, this still requires that the markers are in complete LE and this is a seemingly unrealistic assumption for WGR based on hundreds of thousands or millions of markers.

### Statistical properties of likelihood-based estimates

In the previous section we argued that the statistical parameter $\sigma_{u}^{2}$ agrees with the population parameter $\sigma_{g}^{2}$ only under highly simplified scenarios. Another important question is that of whether estimators derived from potentially misspecified likelihoods (either in likelihood-based or Bayesian estimates) can estimate parameters consistently, meaning that that the estimator converges to the true value of the parameter asymptotically. This will happen, for instance, if the bias and variance of the estimator go to zero as sample size goes to infinity, e.g., [24]).

We focus our study on the G-BLUP procedure due to its widespread use and relative simplicity. In this method, phenotypes are regressed on markers using a linear model of the form $y_{i}\;=\;\sum_{j}x_{ij}b_{j}+\varepsilon_{i}$ where $b_{j}\sim^{iid}N\left(0,\sigma_{b}^{2}\right)$ and $\varepsilon_{j}\sim^{iid}N\left(0,\sigma_{\varepsilon }^{2}\right),$ with marker effects and residuals mutually independent. The model implies the following marginal distribution of phenotypes

$$y\sim N\left(0,G\sigma_{u}^{2}+I\sigma_{\varepsilon }^{2}\right)$$

where *N*(.,.) stands for a multivariate normal distribution, *G* is a genomic-relationship matrix and $\sigma_{u}^{2}$ is a variance parameter. Maximizing the likelihood ($\sigma_{u}^{2}$ and $\sigma_{\varepsilon }^{2}$ are the unknown parameters) function associated with above-expression yields maximum likelihood estimates of variance components and of the proportion of variance explained by the model: $h_{u}^{2}\;=\;\frac{\sigma_{u}^{2}}{\sigma_{u}^{2}+\sigma_{\varepsilon }^{2}}$. Maximization of the likelihood function is facilitated using the eigenvalue decomposition of the *G* matrix; further details about this are given in S3 Text.

Since *G* is computed using markers that are not necessarily QTL or that are in imperfect LD with QTL, the (co)variance patterns of additive effects and, consequently, the likelihood function, can be misspecified [6]. This potentially leads to large finite-sample bias and to inconsistency of estimates, meaning that the genetic parameters may not be well estimated even in large samples. To assess some statistical properties of maximum likelihood estimators (MLE) we conducted two simulations. In both, phenotypes were generated according to the additive QTL model of (Eq 1), *y*_*i*_ = *α′z*_*i*_ + *δ*_*i*_, with QTL effects sampled from a zero-mean normal distribution and from an independent normal distribution with IID residuals, that is $\delta_{i}\sim N(0,\sigma_{\delta \;}^{2})$. Variance parameters were chosen to generate a trait heritability of 0.5. In our first simulation markers and QTL were generated according to a stylized LD pattern. Our second simulation uses real human genotypes.

#### Simulation 1 (simplified LD patterns)

Here, the genome consisted of independent LD blocks. This assumption is not necessarily realistic; however, this setting allows us to determine the value of the genomic heritability parameter and facilitated exploring the effect of the extent of LD (by changing the length of the LD block) on statistical properties of the likelihood-based estimates. To simulate genotypes we: (a) generated haplotypes according to a Markov process, (b) randomly mated haplotypes to generate diploid genotype blocks, and (c) randomly merged genotype blocks to create a genome. Genomes included 50,000 loci; in one set of the simulation scenarios (SB = short blocks) each LD block contained 5 loci and there were 10,000 blocks in mutual LE; in another set of simulation scenarios (LB = long blocks) each block contained 50 loci and there were 1,000 blocks in mutual LE. The LD patterns within blocks were controlled by the parameters of the Markov process used to generate haplotypes. Specifically, in one set of the simulation scenarios (FTP = fixed transition probabilities) the transition probability of the Markov process was the same for all LD blocks; this approach produced blocks with very similar LD patterns. In a second set of simulation scenarios (RTP = random transition probability) the transition probability of each LD block was sampled form a beta distribution. Hence, the LD patterns changed from block to block here. S1 Fig displays the realized LD patterns for each of the simulation scenarios. Full details of the algorithm used to generate genomes are given in the Supplementary Methods section S4 Text.

Once genomes were simulated genetic values were generated according to an additive model. There were 200 QTL with positions chosen at random and with effects sampled from IID normal distributions. In the SB scenarios, 200 blocks were randomly selected out of the 10,000, and a QTL was assigned to a randomly chosen locus within the LD block. In LB scenarios the 200 QTL positions were assigned completely at random within the 50K-loci genome. Full details about the simulation procedure are provided in the Supplementary Methods file S4 Text.

We run a total of 3,000 Monte Carlo (MC) replicates. For each MC replicate, we produced a population of size of 10,000 individuals. The genomes from the entire population were used to calculate population parameters such as genetic, phenotypic and genomic variances using formulae presented previously. From the 10,000 individuals, 1,000 were chosen at random, and data from these were used to estimate trait heritability and genomic heritability by maximum likelihood using a G-BLUP model. The G-BLUP model was fitted using G-matrices computed using: only QTL, QTL and markers in LD with QTL (QTL+MRK.LD), ALL loci, only markers in LD with QTL (MRK.LD), only markers in linkage equilibrium with QTL (MRK.LE) and all markers (MRK.LD+MRK.LE). This resulted in 6 distinct genomic relationship matrices and each yielded an estimate of genomic heritability per MC replicate. According to the theory described in the first section of this article, in the analyses of settings where QTL are in the panel (QTL, QTL+MRK.LD, ALL loci), the genomic and trait heritability parameters coincide. In scenarios without QTL in the panel and including markers in LD with the QTL (MRK.LD, MRK.LD+MRK.LE) the proportion of variance explained is $h_{g}^{2}$. Finally in the scenario including only MRK.LE, the marker panel is not expected to explain any fraction of the phenotypic variance.

#### Simulation 2 (real human genotypes)

For this simulation we used real human genotypes; these reflect LD patterns that are more realistic than those considered in the previous section, at least in humans.

The genotypes used in the simulation were obtained from the type-2 diabetes case-control data set from the Nurses’ Health Study and the Health Professionals Follow-up; both are part of the Gene-Environment Association Studies consortia (GENEVA[25], https://www.genevastudy.org/). This data set was obtained under dbGaP research project #5058 (dbGaP study accession: phs000091.v2.p1); the data set has been designated as “non-human subjects” and approved for general research use by the IRB unit of the University of Alabama at Birmingham (UAB). We used only genotypes of nominally unrelated individuals of Caucasian origin and with less than 5% of missing genotypes. This left 5,000 individuals for the analysis.

The simulation setting was similar to that described in de los Campos et al. [6]: from a set of 400K (K = 1,000), 300K SNP were randomly chosen and designated as markers. From the remaining 100K SNP, 5,000 were chosen and designated as QTL using a sampling method that over-sampled markers with low minor-allele frequency (see Low-MAF scenario in [6] for further details). We run 1,000 MC replicates and, in each MC replicate, 2,500 individuals were randomly sampled from the 5,000 available. QTL effects and model residuals were sampled from IID normal distributions with variance parameters chosen to produce a trait heritability of 0.5, and genetic values and phenotypes were generated according to an additive model as in (Eq 1).

Variance parameters were estimated using G matrices computed with: only QTL loci, only markers (MRK), and by combining markers and QTL (MRK+QTL). According to theory, in the analyses of scenarios QTL and MRK+QTL there is no missing heritability (i.e., the trait and genomic heritability parameters coincide). When only MRK information is used there may be missing heritability but the actual extent is unknown because the population parameters Σ_z_, Σ_zx_ and Σ_z_ cannot be reliably estimated from a sample of 5,000 individuals.

## Results and Discussion

We begin this section by presenting results from the two simulation studies discussed above. This is followed by a discussion of the conceptual and empirical results presented in this article.

### Results from simulation studies

#### Simulation 1 (simplified LD patterns)

S1 Fig shows the squared correlations between the locus at the center of the block (position 3/25 in SB/LB scenarios) and each of the loci in the same block, by position. The average LD patterns are the same across scenarios; however, there is random variation across curves (LD blocks). As desired, the extent of variability in LD patterns is largest in the RTP (random transition probability) scenarios and minimum in the FTP (fixed transition probability) scenarios.

**Fig 1:**
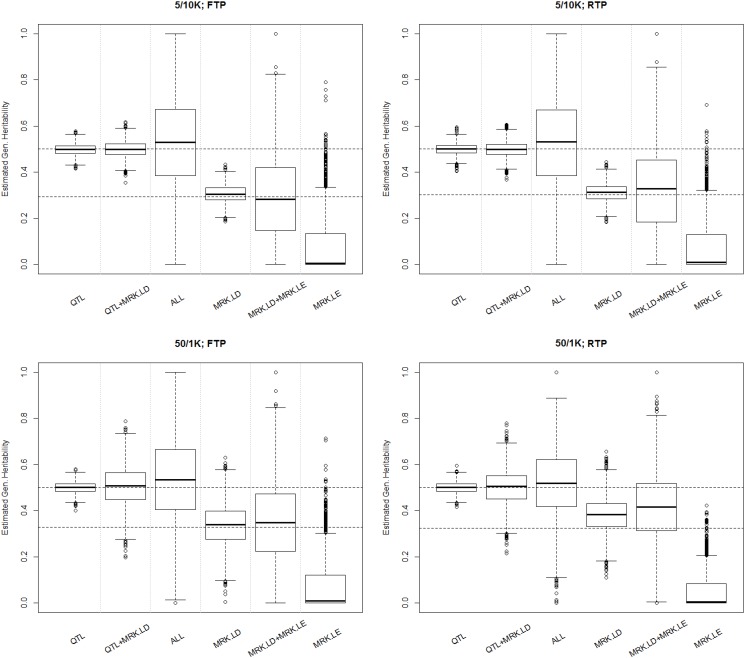
Boxplot of estimated genomic heritability (3,000 MC replicates) by simulation and analysis scenario. Each plot presents results for one simulation scenario (5/10K 10 thousand LD blocks with 5 loci each; 50/1K one thousand LD blocks, each with 50 loci; FTP, ‘fixed transition probability, indicates that the LD patterns were the same across LD blocks, RTP, random transition probability, is a scenario where LD patterns changed between blocks). The labels in the horizontal axis indicate what information was used to compute the G-matrix (QTL = genotypes at causal loci, MRK.LD = markers in LD with QTL, MRK.LE = markers in LE with QTL, ALL = all loci).

Table 1 shows the average (over the 3,000 MC replicates) estimates of genomic heritability, and Fig 1 displays box plots of estimates by scenario and method. Columns 3–4 of Table 1 provide the true value of the population parameters and columns 5–10 provide the average MC estimates and the corresponding SEs, by scenario and model. Trait heritability was 0.5, and genomic heritability ranged from values close to 0.30 in SB scenarios and slightly higher .325-.328 in LB scenarios. Columns 5–7 give results of analyses from scenarios where QTL were included in the marker panel; hence, without missing heritability. When only QTL genotypes were used to compute the G-matrix, the average estimated genomic heritability was 0.5 and the estimates were rather precise (SE 0.024). This suggests that ML estimation with this sample size yielded estimates with no detectable bias, that is, when the model holds. When QTL and MRK in LD with QTL were used to compute G, the ML estimate of genomic heritability was seemingly unbiased in the SB scenario, and had a very small upward bias in the LB scenarios. Columns 8–9 of Table 1 show results obtained when G was computed using MRK.LD and without using QTL. In this scenario there is missing heritability: the genomic heritability is smaller than the trait heritability. In the SB-FTP scenario using MRK.LD genotypes only, the genomic heritability was estimated almost without bias. However, in all other simulation scenarios (SB-RTP, LB-FTP and LB-RTP) there was a sizable upward bias in estimates of genomic heritability. The bias was accentuated when MRK.LE were added to MRK.LD when computing G. These results are in line with what we observed in the analysis with ALL loci, and suggest that adding a large number of markers in LE with QTL in the analysis induces bias and increases the sampling variance of estimates.

**Table 1:** Mean (SD) of estimates of genomic heritability by simulation scenario (rows) and information used for analysis.

LD Block	Trans. Prob.	Parameter Values		Average (SE) Maximum Likelihood Estimate					
		*h* ^2^	$h_{G}^{2}$	QTL	QTL+ MRK.LD	ALL	MRK.LD	MRK.LE+MR.LD	MRK.LE
**Short**	Fixed	.500	.295	.498 (.024)	.499 (.035)	.536 (.225)	.305 (.036)	.289 (.191)	.078 (.113)
	Rand.	.500	.303	.500 (.024)	.499 (.033)	.535 (.214)	.312 (.034)	.324 (.187)	.075 (.105)
**Long**	Fixed	.500	.328	.500 (.024)	.505 (.085)	.543 (.210)	.337 (.087)	.353 (.189)	.071 (.100)
	Rand.	.500	.325	.500 (.024)	.502 (.074)	.518 (.152)	.381 (.071)	.415 (.144)	.051 (.071)

*h* ^2^: trait heritability; $h_{G}^{2}$: genomic heritability; Short/Long refer to the length of the LD blocks. Fixed/Rand define whether the LD patterns were the same (Fixed) or varied (Rand.) between blocks (Rand). QTL (only QTL), QTL+MRK.LD (QTL and markers in LD with QTL), ALL (all loci), MRK.LD (only markers in LD with QTL), MRK.LD+MRK.LE (only markers, no QTL) and MRK.LE (only markers in LE with QTL) were used to compute the genomic relationship matrix.

#### Simulation 2 (real human genotypes)

**Fig 2:**
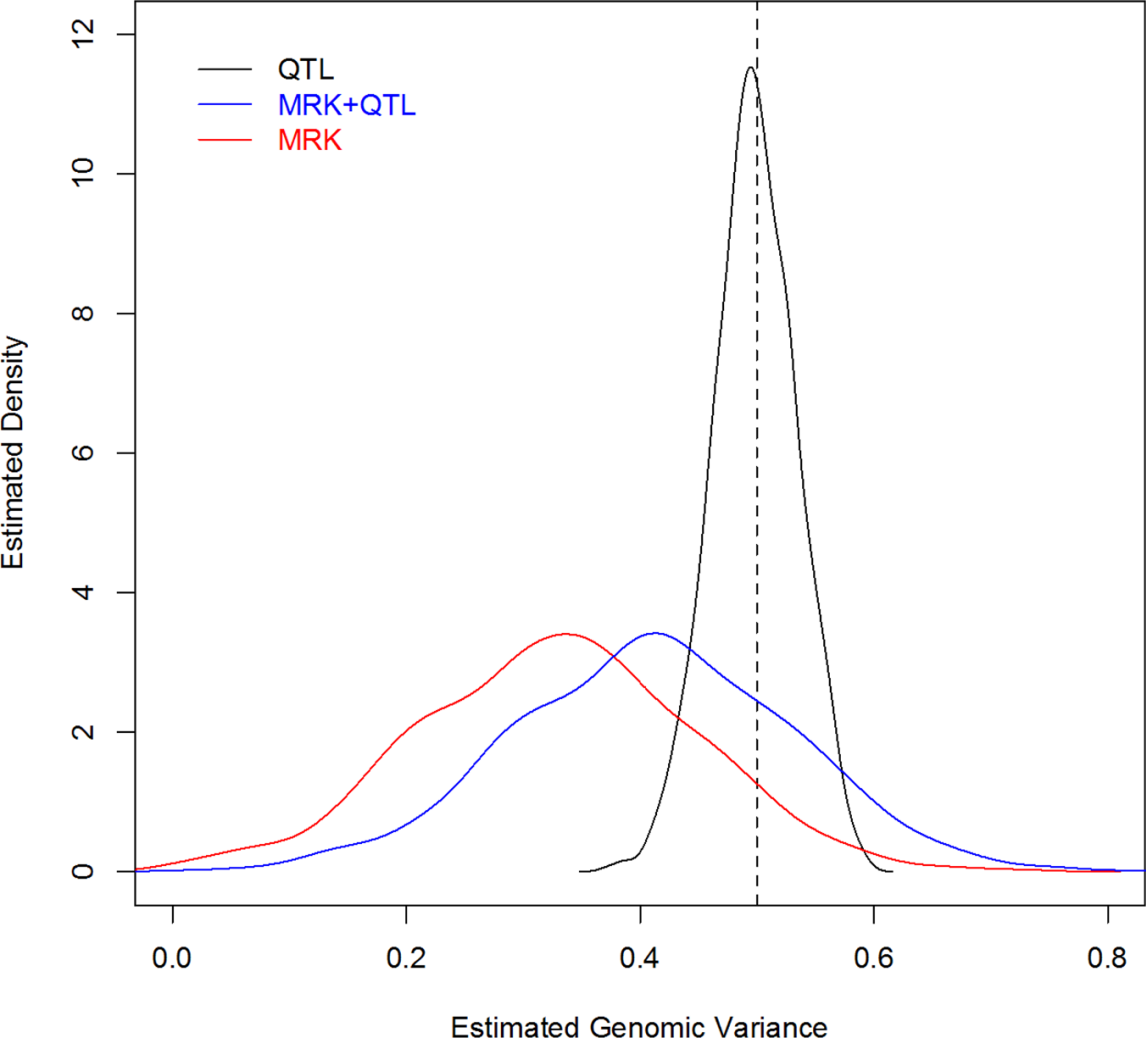
Density plot of estimated genomic heritability (1,000 MC replicates) by analysis scenario (Simulation 2). The vertical dashed line gives the simulated heritability (QTL, MRK, MRK+QTL indicate whether QTL genotypes, or marker genotypes, MRK, or both, MRK+QTL, were used to compute genomic relationships).

For each MC replicate a G-BLUP model was fitted to the 2,500 records, using a G matrix computed from: QTL genotypes, MRK, and QTL+MRK. Fig 2 displays the estimated density plots for each case. In the analysis using only QTL information the average estimated genomic heritability (.498) was very close to the trait heritability (.5); the estimated 90% confidence interval ranged from .438 to .558. The analysis with markers only showed an average estimated genomic heritability of .328, that is an estimated extent of missing heritability of 34%, similar to that reported by de los Campos et al. (2013) [6] who analyzed data simulated with a similar but not identical scheme. The sampling variance of the estimator was very large, with a 90% CI for the estimated genomic heritability with MRK ranging from .136 to .517. This indicates that, when constructing G, adding markers that are in imperfect LD with QTL adds considerable uncertainty to estimates of variance components. Finally, the distribution of the estimates of genomic heritability obtained with markers and QTL was similar to that obtained with markers only, but shifted to the right, with a mean equal to .411. In this scenario, as stated, the trait and genomic heritability parameter coincide (i.e., there is no missing heritability); therefore, the result indicates that G-BLUP leads to a downward-biased estimate of the genomic heritability and incorrectly suggests the existence of missing heritability when there is not.

## Discussion

The first attempts at using molecular markers for assessment of kinship and for estimation of trait heritability originate in the analysis of natural populations more than half a century ago [9–12,26–28]. Several of these studies indicated that, for distant relatives, the proportion of allele sharing varies substantially among chromosome segments and, consequently, the accuracy of estimators of relatedness using markers is low unless the number of markers is very large. The advent of dense genome-wide SNP data brought a revival of the topic and made it possible to obtain inferences of genetic parameters using nominally unrelated individuals [4].

In the literature on genomic analysis of complex traits, parameters such as the genomic heritability have been defined based on the model used for data analysis [4–6,29]. This approach has two potential pitfalls. Firstly, there is a key difference between the way genotypes and their effects are dealt with and interpreted in quantitative genetics and in the random regression models used for data analysis. In quantitative genetics genetic variance stems from variation of allele content at QTL loci, and QTL effects are fixed quantities at a given time in a population [15–17]. This is in contrast with the statistical models used for data analysis, where marker genotypes are treated as fixed and effects are regarded as random. In the latter models variance stems from uncertainty about the unknown effects and this bears no immediate connection with the concept of genetic variance. This results in a tenuous link between these parameters and those from quantitative genetics theory. Secondly, because patterns of allele sharing vary across the genome and because markers are typically in imperfect LD with QTL, the patterns of variance and covariance at QTL and at markers can be very different. This is especially important for distantly related individuals [6]. Under these circumstances the likelihood function of marker-based models may largely misrepresent the underlying data generating process, leading to potential inferential problems (e.g., inconsistency as well as finite sample bias and very low precision of estimates).

***A first contribution of this article*** is to provide ***theory***, framed within the principles of quantitative genetics, proposing precise definitions of parameters of the instrumental model (marker effects, genomic variance) at the population level. A few important results emerge from the definitions and derivations presented in this article.

1. From a quantitative genetics perspective ***QTL marker and effects*** (see expressions (2) and (5), respectively) ***are fixed population quantities*** and not random variables. Although mutation, the process that gives rise to new variants and their effects, can be viewed as random, at any given time in a population, the regressions of genetic values on allele content at QTL (*α*) take on a given set of values. What makes individuals genetically different is the fact that they carry different alleles at QTL loci. Therefore, genetic and genomic variance stems from variation of allele content at QTL and at markers, respectively, (see expression (3) and (6)) and not from uncertainty or randomness about QTL or marker effects. This seems at odds with definitions of genomic variance based on random effects or with Bayesian models where marker genotypes are treated as “fixed” and marker effects as random variables. From a Bayesian perspective it makes perfect sense to implement regressions conditioning on markers and with marker effects treated as random variables because marker genotypes are observable and marker effects are unknown, i.e., a posterior distribution is conditional on all pertinent observables. However, we question the use of these Bayesian models for definition of genetic parameters. The variance parameters of a Bayesian model, aimed to reflect uncertainty about marker effects, bear little conceptual connection with the concept of genetic variance [23].
2. ***Marker effects are linear combination of QTL effects*** (see expression (5)). With high marker density multiple markers are likely to track variance from the same QTL. This ***questions the treatment of marker effects as independent random variables***. For example, if, from a Bayesian perspective, QTL effects were viewed as IID draws from a normal distribution, then it follows from expression (5) that marker effects are MVN distributed with null mean and covariance matrix $Cov\left(\beta \right)\propto {\Sigma_{x}^{-1}\Sigma }_{xz}\Sigma_{zx}\Sigma_{x}^{-1}$. Some of the off-diagonals of ${\Sigma_{x}^{-1}\Sigma }_{xz}\Sigma_{zx}\Sigma_{x}^{-1}$ may be null; however, assuming *a priori* that all the covariances are null and that all the diagonal entries are the same ignores the fact that multiple markers can track variance from the same QTL. Determining the correct covariance function is not possible because in almost all cases the positions of QTL are unknown and the coding of markers is arbitrary, thus precluding determination of Σ_xz_. However attempts can be made at incorporating LD information in the prior distribution in some sensible manner. For instance, [30] proposed incorporating LD information in the prior density assigned to marker effects using parametric covariance functions (e.g., auto-regressive). A limitation of this approach is that the sign of the marker effect can be arbitrarily changed by recoding the marker. A solution to this problem is to incorporate LD information at the level of the variances of the maker effects, as proposed by Yang and Tempelman [31]; this induces smoothness on the size of the effects without imposing restrictions on the sign.
3. The recognition that marker effects are linear combinations of QTL effects has a second important consequence: ***LD between markers plays a central role in the determination of genomic variances*** (see expression (6)). It is only under very idealized (and seemingly unrealistic) conditions that one can decompose the total genetic and genomic variance into marker-specific components. On the other hand, in the linear regression models commonly used for data analysis marker effects are treated as IID random variables and genotypes as fixed quantities, leading to a decomposition of the genomic variance that does not involve LD. Depending on the patterns of LD between markers, ignoring LD may lead to under or over estimation of the genomic variance. This illustrates again the weak connection existing between the genomic variance as a population parameter and the parameters that are usually defined based on statistical assumptions that are adopted for computational or other reasons, but without reference to an underlying QTL model.
4. Intuitively, it should clear that ***if causal variants are in the marker panel*, *there should be no missing heritability***. Here, we presented a formal proof of this intuitive expectation: we showed that when causal variants are included in the marker panel the trait and genomic heritability parameters coincide. The implication is that with sequence data there should be no missing heritability at the population level. Empirically, however, estimates may suggest missing heritability; if this happens, this would be reflective of shortcomings of the estimator chosen.

### Estimation of genomic variance using G-BLUP and maximum likelihood

Above, we discussed conceptual problems emerging when genetic parameters are defined based on assumptions of statistical models that are not in line with fundamental quantitative genetic concepts. A second problem arises when estimation of these parameters is based on markers and not on QTL-genotypes. Under regularity conditions, maximum likelihood estimates are asymptotically unbiased ([24]). However, consistency cannot be guaranteed unless the likelihood is correctly specified and, even if the estimator happens to be consistent, misspecified likelihoods can induce sizable finite-sample bias. The proportion of allele sharing at any given set of loci can be viewed as a random variable with expected value given by twice the kinship coefficient between the individuals (derived from the full pedigree and with additional assumptions such as absence of mutation and no selection) and random variation given by the effects of Mendelian sampling [32]. If a large number of markers segregate independently from the QTL the proportion of allele sharing at markers and at QTL can be very different (e.g., [6]). This is particularly important for pairs of individuals sharing short chromosome segments (e.g., distantly related individuals from populations with large effective population size). Under these conditions a likelihood, constructed based on proportions of allele sharing at markers, can be largely misspecified and consistency may not hold. Of course, this does not necessarily imply that estimates would be inconsistent or have noticeable finite-sample biases; it just poses a caveat: one needs to be careful with use of estimates derived from models based on misspecified likelihoods, as it is most probably the case for marker-based model for whole-genome inference.

There are at least two cases where a likelihood function based on markers will not be largely misspecified. The first one is when patterns of allele sharing at markers and at QTL are very similar; this can occur if markers are in tight LD with QTL, and also with data from close relatives. A second case is when the components of genetic values that cannot be explained by markers (ξ_i_ in expression (4)) are IID, in which case the likelihood will correctly represent the (co)variance structure of the data. In these two cases the parameters of the instrumental model (e.g., $h_{u}^{2}$) could be inferred consistently. Unfortunately, in general there is no good reason to believe that the ξ_i_′s are IID and therefore the likelihood function will typically be misspecified.

Goddard et al. [33] suggested a way of computing the marker-based genomic relationship matrix which, the authors argue, would have the property that the expected value of the proportion of allele sharing at QTL (*G*_*QTL*_) given the realized proportions of allele sharing at markers (*G*_*MRK*_) is *G*_*MRK*_, that is *E*[*G*_*QTL*_|*G*_*MRK*_] = *G*_*MRK*_. However, even if this property were to hold, this does not imply that estimates of variance components derived using *G*_*MRK*_ would be unbiased. Indeed, in each sample *G*_*QTL*_ can differ from *G*_*MRK*_; if the differences in the proportion of allele sharing at markers and at QTL are large enough, misspecification of the likelihood function due to the fact that we use *G*_*MRK*_ instead of *G*_*QTL*_, can induce a systematic bias.

Recently Jiang et al. [34] examined large sample properties of REML estimators of variance parameters of a marker-based regression ($\sigma_{\varepsilon }^{2},\;\sigma_{u}^{2}$) and concluded that, under certain conditions, the REML estimators can converge in probability to the true value of the statistical parameters, this being the case even if the likelihood is misspecified. The proof of this result is based on three key assumptions: (a) the model used to estimate parameters, what we here call the instrumental model, includes all QTL genotypes plus a number of markers with no effect, (b) all the covariates used in the model (i.e., both QTLs and markers) are mutually independent, and (c) the number of QLT is not too small relative to the number of markers. In this specific setting the authors prove consistency of the REML estimator of the error variance and the convergence in probability of the REML estimator of the statistical parameter ${\;\sigma }_{u}^{2}$. This result, which the authors verified in simulations, indicates some robustness of the REML estimator. On the other hand, in our study we detected several cases where misspecified likelihoods produced sizable finite-sample bias, this occurring in settings where ML estimates derived from correctly specified likelihoods were seemingly unbiased. However, there are important differences between the study of Jiang et al. [34] and ours. Firstly, Jiang et al. [34] focused on estimation of the statistical parameters of the instrumental model ($\sigma_{\varepsilon }^{2},\;\sigma_{u}^{2}$) while here we addressed estimation of parameters defined from a quantitative genetics perspective ($\sigma_{\varepsilon }^{2},\;\sigma_{g}^{2}$) and, as we discussed here, $\sigma_{u}^{2}$ and $\sigma_{g}^{2}$ coincide only under very stylized conditions (e.g., complete LE between loci, a condition assumed by Jiang et al. [34] which we did not adopt here). Secondly, in an attempt to resemble what one encounters with modern genomic data, our simulations used a ratio between the number of QTL relative to the total number of loci that is relatively small (200/50K in simulation 1, and 5/305 in simulation 2). Importantly, Jiang et al. [34] indicate that the asymptotic results presented in their study involve approximations that may not hold when the ratio of number of QTL relative to the total number of loci is close to zero. Thirdly, our simulations incorporated LD (either LD blocks in simulation 1 or LD patterns realized in real human genotypes in simulation 2); therefore, in our case loci were not in mutual LE as assumed in Jiang et al. [34]. Fourthly, in the simulation used in [34] the ratio between the total number of loci (both marker and QTL) and sample size was 10; in our simulations this ratio was 50 in simulation 1 and approximately 60 in simulation 2. We adopted these settings because with modern genomic data the ratio between the number of markers and sample size is expected to be large. In the light of the evidence presented here it seems clear that further studies are needed to characterize the finite sample properties of REML estimators derived using misspecified likelihoods in settings that resemble the conditions encountered in the analysis of real genomes.

Simulation studies using real human genotypes, including the one presented here, indicate that estimates of $h_{u}^{2}$ based on the G-BLUP model incorporating both markers and all QTL genotypes are lower than the trait heritability of the trait ${(h}_{}^{2})$. This reinforces the idea that GBLUP may not lead to unbiased estimates of the genomic heritability ($h_{g}^{2}$) because when all QTL are included in the marker panel $h_{g}^{2}\;=\;h_{}^{2}$. Speed et al. [5] argued that a main reason for incorrect estimation is that LD is ignored in the computation of the G matrix. These authors suggested an alternative method for computing G that resulted in estimates of genomic heritability closer to the simulated trait heritability. However, in a follow up discussion [35,36] presented alternative simulations scenarios where the method of Speed et al. [5] yielded biased estimates. All in all, this suggests that the appropriate choice of method used for computing G may depend on the genetic architecture of the trait, a feature that is typically unknown, even if attention is restricted to additive gene action only. From our perspective, the main problem does not reside in the manner the G matrix is computed but, rather, in the use of massive numbers of markers that are in LE with QTL. The use of such data increases the sampling variance of estimates and may cause large finite-sample bias and lack of consistency.

### Estimation of genomic heritability using WGR models

We have argued that the instrumental parameter $h_{u}^{2}$ may not provide a good representation of the genomic heritability ($h_{g}^{2})$; an important reason for the difference between these two parameters is that while LD plays a role in the determination of $\sigma_{g}^{2}$ associations among marker genotypes are ignored in the definition of $\sigma_{u}^{2}$. An alternative approach that accounts for multi-locus LD, could be based on the sample-variance of the true genomic values [37] that is σ~g2 = n-1∑i = 1n xi'β-x¯i'β2  where ${\bar{x}}_{i}$ represents the average genotype. Of course, marker effects are unknown; however as suggested by Sorensen et al. [37] one could infer ${\tilde{\sigma }}_{g}^{2}$ in a Bayesian setting by evaluating ${\tilde{\sigma }}_{g}^{2}$ using samples from the posterior distribution of marker effects. The parameter ${\tilde{\sigma }}_{g}^{2}$ accounts for LD between markers in a very specific manner: ${\tilde{\sigma }}_{g}^{2}$ becomes equal to the genomic variance parameter, $\sigma_{g}^{2}\;=\;\beta^{'}\Sigma_{x}\beta$ (see expression 7), with Σ_*x*_ replaced by its method-of-moments estimator *X′ Xn* ^-1^. Indeed, when genotypes are centered,

σ~g2 = n-1∑i = 1n xi'β2  = n-1∑i = 1nβxi xi'β  = n-1β'∑i = 1nxi xi' β = β'n-1X'Xβ.

### Analysis and prediction of complex traits using Whole-Genome Regressions

Complex traits are possibly affected by large numbers of small-effect QTL and the analysis of such traits requires fitting a large number of variants concurrently using a WGR approach such as the one proposed by [1]. Close relatives share long chromosome segments and, under these circumstances, the patterns of allele sharing at markers and at QTL are very similar. This leads to high prediction accuracy and very small bias in genomic heritability estimates. When markers and QTL co-segregate, variable selection does not seem to be needed [6]. On the other hand with distantly related individuals, the addition of large numbers of markers that are in LE with QTL can lead to incorrect specification of genomic relationships and this can result in potential inconsistencies of estimates of genomic heritability. We do not question the use of WGR for analysis of complex traits and as a prediction machine. Rather, we warn about problems arising when these methods are used for inferences. In our opinion this problem has been overlooked and oversimplified, and further research is needed to understand if and under what circumstances WGRs such as the G-BLUP can be used to correctly assess the true proportion of variance that can be explained by a regression on markers in the population.

## Supporting Information

S1 Text Proof of rotation invariance of the genomic heritability. (DOCX)

S2 Text Marker effects and missing heritability when all “causal variants” are in the marker Panel. (DOCX)

S3 Text Maximum Likelihood Estimation of Genomic Heritability with the G-BLUP model (DOCX)

S4 Text Simulation of genotypes with simplified covariance structures. (DOCX)

S1 Fig Squared correlations between genotypes at different loci within a block with the genotype at the mid-point of the block, by simulation scenario (SB/LB = short/long LD block, 5/50 loci per LD block; FTB/RTB = fixed/random transition probability). The grey lines give the realized LD patterns for different blocks and the red dashed line gives the average (across block) LD pattern.
(PDF)
