# Supplementary material for: Genomic Heritability: What Is It?
Source: PLoS Genet. 2015 May 5;11(5):e1005048. doi: 10.1371/journal.pgen.1005048 (PMC4420472; doi:10.1371/journal.pgen.1005048)
Supplement: S1 Text — (DOCX) [file pgen.1005048.s001.docx]

## Supplementary Methods I:

## Proof of the rotation invariance property of the genomic heritability

We show here that the genetic and genomic variances (expressions 3 and 6, respectively) are invariant under linear transformations of genotypes; therefore, these parameters, and functions thereof such as the trait heritability and the genomic heritability, do not depend on the way genotypes are coded. To see this consider arbitrary linear transformations (recoding is a full-rank linear transformation) of the QTL and marker genotypes of the form: $\tilde{z}_{i}=\tau_{z}+T_{z}z_{i}$ and $\tilde{x}_{i}=\tau_{x}+T_{x}x_{i}$. After transformation, the relevant covariance matrices become $\Sigma_{\tilde{z}}=T_{z}\Sigma_{z}T_{z}'$, $\Sigma_{\tilde{x}}=T_{x}\Sigma_{x}T_{x}^{'}$, $\Sigma_{\tilde{z}g}=T_{z}\Sigma_{zg}$*,* $\Sigma_{\tilde{x}z}=T_{x}\Sigma_{xz}$ and $\Sigma_{\tilde{x}\tilde{z}}=T_{\tilde{x}}\Sigma_{xz}T_{\tilde{z}}'$. Therefore, the effects of the transformed QTL and marker genotypes become

$\tilde{\alpha}{=\Sigma_{\tilde{z}}^{-1}\Sigma}_{\tilde{z}g}= {{T_{z}}^{'-1}\Sigma}_{z}^{-1}T_{z}^{-1}T_{z}\Sigma_{zg}= {{T_{z}'}^{-1}\Sigma}_{z}^{-1}\Sigma_{zg}={T_{z}}^{'-1}\alpha$

and

$\tilde{\beta}=Var\left( \tilde{x}_{i} \right)^{-1}Cov\left( \tilde{x}_{i},\tilde{z}_{i}'\tilde{\alpha} \right)=\left( {T_{x}}^{'-1}\Sigma_{x}^{-1} {T_{x}}^{-1} \right) \left( T_{x}\Sigma_{xz}T_{\tilde{z}}' \right) {T_{z}}^{'-1}\alpha={T_{x}}^{'-1}\Sigma_{x}^{-1}\Sigma_{xz}\alpha={T_{x}}^{'-1}\beta$ ,

respectively. Therefore, the additive and genomic variance become

$$Var\left( \tilde{\alpha}^{'}\tilde{z}_{i} \right)={{\alpha'T}_{z}}^{-1}{{T_{z}\Sigma_{z}T_{z}'T}_{z}}^{'-1}\alpha=\alpha^{'}\Sigma_{z}\alpha$$

and

$Var\left( \tilde{\beta}'\tilde{x}_{i} \right)=\beta'{T_{x}}^{-1}T_{x}\Sigma_{x}T_{x}^{'}{T_{x}}^{'-1}\beta$=$\beta'\Sigma_{x}\beta$

This means that while the sign of the effects is arbitrary (i.e., it depends on how genotypes are coded), the variance parameters are invariant with respect to any arbitrary linear transformation, including recoding.
