# Supplementary material for: Genomic Heritability: What Is It?
Source: PLoS Genet. 2015 May 5;11(5):e1005048. doi: 10.1371/journal.pgen.1005048 (PMC4420472; doi:10.1371/journal.pgen.1005048)
Supplement: S2 Text — (DOCX) [file pgen.1005048.s002.docx]

## Supplementary Methods II:

## Marker effects and missing heritability when all “causal variants” are in the marker Panel

In this section we show that when all “causal variants” are part of the marker panel the vector of marker effects satisfies: $\beta_{j}=\left\{ \alpha_{j} if x_{j} is a QTL ;0 otherwise \right\}$; consequently the genomic variance equals the additive variance and there is no missing heritability, as one would expect. We prove this using standard results on the inverse of a partitioned matrix. To this end we partition the markers into the set of markers that are not QTL (set 1) and markers that are QTL (set 2); therefore,$x_{i}=\left( x_{1i}',x_{2i}' \right)'=\left( x_{1i}',z_{i}' \right)'$ . Accordingly, the covariance matrix of marker genotypes is partitioned as follows:

$$\Sigma_{x}=\left[ \begin{matrix} \Sigma_{11} & \Sigma_{12} \\ \Sigma_{21} & \Sigma_{22} \end{matrix} \right]$$

where $\Sigma_{11}$ and $\Sigma_{22}$ represent (co)variance matrices pertaining to markers that are not QTL and of markers that are causal, respectively, and $\Sigma_{21}=\Sigma_{12}^{'}$ represents the matrix describing covariances between markers that are not QTL and those that are causal. Further$\Sigma_{xz}=\left[ \begin{aligned} Cov\left( x_{1i},z_{i}' \right) \\ Cov\left( x_{2i},z_{i}' \right) \end{aligned} \right]=\left[ \begin{aligned} \Sigma_{12} \\ \Sigma_{22} \end{aligned} \right]$; therefore,

$$\beta={\Sigma_{x}^{-1}\Sigma}_{xz}\alpha=\left[ \begin{matrix} \Sigma_{11} & \Sigma_{12} \\ \Sigma_{21} & \Sigma_{22} \end{matrix} \right]^{-1}\left[ \begin{aligned} \Sigma_{12} \\ \Sigma_{22} \end{aligned} \right]\alpha$$

Using a standard result on the inverse of a partitioned matrix we have:

$$\Sigma_{x}^{-1}=\left[ \begin{matrix} \left( \Sigma_{11}-\Sigma_{12}\Sigma_{22}^{-1}\Sigma_{21} \right)^{-1} & -\left( \Sigma_{11}-\Sigma_{12}\Sigma_{22}^{-1}\Sigma_{21} \right)^{-1}\Sigma_{12}\Sigma_{22}^{-1} \\ -\left( \Sigma_{22}-\Sigma_{21}\Sigma_{11}^{-1}\Sigma_{12} \right)^{-1}\Sigma_{21}\Sigma_{11}^{-1} & \left( \Sigma_{22}-\Sigma_{21}\Sigma_{11}^{-1}\Sigma_{12} \right)^{-1} \end{matrix} \right]$$

Therefore,

$$\Sigma_{x}^{-1}\Sigma_{xz}=\left[ \begin{matrix} \left( \Sigma_{11}-\Sigma_{12}\Sigma_{22}^{-1}\Sigma_{21} \right)^{-1} & -\left( \Sigma_{11}-\Sigma_{12}\Sigma_{22}^{-1}\Sigma_{21} \right)^{-1}\Sigma_{12}\Sigma_{22}^{-1} \\ -\left( \Sigma_{22}-\Sigma_{21}\Sigma_{11}^{-1}\Sigma_{12} \right)^{-1}\Sigma_{21}\Sigma_{11}^{-1} & \left( \Sigma_{22}-\Sigma_{21}\Sigma_{11}^{-1}\Sigma_{12} \right)^{-1} \end{matrix} \right]\left[ \begin{aligned} \Sigma_{12} \\ \Sigma_{22} \end{aligned} \right]$$

$=\left[ \begin{aligned} \left( \Sigma_{11}-\Sigma_{12}\Sigma_{22}^{-1}\Sigma_{21} \right)^{-1}\Sigma_{12}-\left( \Sigma_{11}-\Sigma_{12}\Sigma_{22}^{-1}\Sigma_{21} \right)^{-1}\Sigma_{12}\Sigma_{22}^{-1}\Sigma_{22} \\ -\left( \Sigma_{22}-\Sigma_{21}\Sigma_{11}^{-1}\Sigma_{12} \right)^{-1}\Sigma_{21}\Sigma_{11}^{-1}\Sigma_{12}+\left( \Sigma_{22}-\Sigma_{21}\Sigma_{11}^{-1}\Sigma_{12} \right)^{-1}\Sigma_{22} \end{aligned} \right]$

$=\left[ \begin{aligned} 0 \\ \left( \Sigma_{22}-\Sigma_{21}\Sigma_{11}^{-1}\Sigma_{12} \right)^{-1}\left( \Sigma_{22}-\Sigma_{21}\Sigma_{11}^{-1}\Sigma_{12} \right) \end{aligned} \right]=\left[ \begin{aligned} 0 \\ I \end{aligned} \right]$

Consequently

$$\beta={\Sigma_{x}^{-1}\Sigma}_{xz}\alpha=\left[ \begin{aligned} 0 \\ I \end{aligned} \right]\alpha=\left[ \begin{aligned} 0 \\ \alpha\end{aligned} \right]$$

In conclusion, when QTL are part of the marker panels, then marker effects are either zero (this for markers that are not QTL), or equal to the effect of allele substitution of the QTL (in case of markers that are QTL). Using (6), and the result derived above, $\Sigma_{x}^{-1}\Sigma_{xz}=\left[ \begin{aligned} 0 \\ I \end{aligned} \right]$, and the genomic variance becomes

$\alpha'\Sigma_{zx}\Sigma_{x}^{-1}\Sigma_{xz}\alpha=\alpha'\Sigma_{zx}\left[ \begin{aligned} 0 \\ I \end{aligned} \right]\alpha$

$=\alpha'\left[ \begin{matrix} Cov\left( z_{i},x_{1i}' \right) & Cov\left( z_{i},x_{2i}' \right) \end{matrix} \right]\left[ \begin{aligned} 0 \\ I \end{aligned} \right]\alpha$

$=\alpha'Cov\left( z_{i},x_{2i}' \right)\alpha$

$=\alpha'\Sigma_{z}\alpha$

The results imply that when all QTL are part of the marker panel the genomic variance equals the additive variance and there is no missing heritability, as one would expect.
