# Supplementary material for: Genomic Heritability: What Is It?
Source: PLoS Genet. 2015 May 5;11(5):e1005048. doi: 10.1371/journal.pgen.1005048 (PMC4420472; doi:10.1371/journal.pgen.1005048)
Supplement: S3 Text — (DOCX) [file pgen.1005048.s003.docx]

## Supplementary Methods III:

## Maximum Likelihood Estimation of Genomic Heritability with the G-BLUP model

Variance components were estimated using maximum likelihood (ML). Genotypes and phenotypes were centered at the population level. The model for data analysis was $y=u+\varepsilon$ where $y, u,\varepsilon$ are all n-dimensional vectors of phenotypes, genomic values and model residuals, respectively. Genomic values have distribution $u\sim N\left( 0,G\sigma_{u}^{2} \right),$ independent of $\varepsilon\sim N(0,I\sigma_{\varepsilon}^{2})$, implying that $y\sim MVN\left[ 0,P \right]$, where $P={G\sigma}_{u}^{2}+I\sigma_{\varepsilon}^{2}$. Here $G$ represents a genomic relationship matrix and $\sigma_{u}^{2}$ and $\sigma_{\varepsilon}^{2}$ are the parameters to be estimated. The likelihood function is

$L\left( \sigma_{\varepsilon}^{2},\sigma_{u}^{2}|y \right) \propto\left\| P \right\|^{-\frac{1}{2}}Exp\{-\frac{y^{'}P^{-1}y}{2}\}$. (S2.1)

For ease of computation we represent the likelihood function using the eigenvectors and eigenvalues of G. This representation has been discussed before elsewhere [1-3]. We describe the implementation in the context of a purely random effects model. Extensions to models involving fixed and random effects based on restricted (or residual) likelihood approaches are discussed by Zhou and Stephens [2].

Eigenvalue decomposition

The matrix G is symmetric; therefore all its eigenvalues are real. The eigenvalue decomposition (EVD) of G is given by $UDU'$ . Here, $U$ is a matrix whose columns are the eigenvectors of G and $D=Diag\{d_{i}\}$ is a diagonal matrix containing the eigenvalues of G. Because U is orthonormal U’U*=I;* furthermore since G is symmetric $U^{'}U=UU^{'}=I$

Replacing the identity matrix in the right-hand-side of $P={G\sigma}_{u}^{2}+I\sigma_{\varepsilon}^{2}$ with $U{IU}^{'}$, we get

$P=\sigma_{\varepsilon}^{2}\left[ UDU^{'}\frac{\sigma_{u}^{2}}{\sigma_{\varepsilon}^{2}}+UU^{'} \right]=\sigma_{\epsilon}^{2}\left[ U\left\{ D\lambda+I \right\}U^{'} \right]$ (S2.2)

$$=\sigma_{\varepsilon}^{2}U\tilde{D}U'$$

where $\lambda=\frac{\sigma_{u}^{2}}{\sigma_{\varepsilon}^{2}}$ and $\tilde{D}=Diag\{d_{i}\lambda+1\}$.

Eigenvalue decomposition and determinants

The determinant of a square matrix equals the product of its eigenvalues, and the determinant of the product of two matrices is the product of its determinants; therefore:

$\left\| P \right\|= \left\| \sigma_{\varepsilon}^{2}U\tilde{D}U' \right\|=\left\| I\sigma_{\varepsilon}^{2}U\tilde{D}U' \right\|=\sigma_{\varepsilon}^{2n}\prod_{i=1} \left( d_{i}\lambda+1 \right)$ (S2.3)

Inverse of the phenotypic co-variance matrix

The evaluation of the likelihood function requires computing the inverse of P, using (S2.2) we have

$${P=\sigma}_{\varepsilon}^{2}U\tilde{D}U'$$

and its inverse is

$${P^{-1}=\sigma}_{\varepsilon}^{-2}\left[ U\tilde{D}U^{'} \right]^{-1}$$

$${=\sigma}_{\varepsilon}^{-2}{U^{'}}^{-1}\tilde{D}^{-1}U^{-1}$$

$${=\sigma}_{\varepsilon}^{-2}{U^{'}}^{-1}\tilde{D}^{-1}U^{-1}$$

${=\sigma}_{\varepsilon}^{-2}U\tilde{D}^{-1}U'$ (S2.4)

Therefore, the eigenvectors of the inverse of P are the same as those of P, and the eigenvalues of the inverse of P are simply the reciprocals of those of P.

Using (S2.3) and (S2.4) in S2.1, the likelihood function is

$\left( \sigma_{\varepsilon}^{2},\sigma_{u}^{2}|y \right) \propto\left\{ \sigma_{\varepsilon}^{2n}\prod_{i=1} \left( d_{i}\lambda+1 \right) \right\}^{-\frac{1}{2}}Exp\left\{ -\frac{y^{'}U\tilde{D}^{-1}U^{'}y}{{2\sigma}_{\varepsilon}^{2}} \right\}$

$L\left( \sigma_{\varepsilon}^{2},\sigma_{u}^{2}|y \right) \propto\left\{ \sigma_{\varepsilon}^{2n}\prod_{i=1} \left( d_{i}\lambda+1 \right) \right\}^{-\frac{1}{2}}Exp\left\{ -\frac{\tilde{y}\tilde{'D}^{-1}\tilde{y}}{{2\sigma}_{\varepsilon}^{2}} \right\}$

where ${\tilde{y}=U}^{'}y$. Therefore the log likelihood is

$\log\left\{ L\left( \sigma_{\varepsilon}^{2},\sigma_{u}^{2}|y \right) \right\} \propto-\frac{1}{2}\left\{ n\times log\left( \sigma_{\varepsilon}^{2} \right)+\frac{1}{2}\sum_{i} log\left( d_{i}\lambda+1 \right)+\frac{\tilde{y}'\tilde{D}^{-1}\tilde{y}}{\sigma_{\varepsilon}^{2}} \right\}$

and

$$-2log \left\{ L\left( \sigma_{\varepsilon}^{2},\sigma_{u}^{2}|y \right) \right\} \propto n\times log\left( \sigma_{\varepsilon}^{2} \right)+\frac{1}{2}\sum_{i} log\left( d_{i}\lambda+1 \right)+\frac{\tilde{y}'\tilde{D}^{-1}\tilde{y}}{\sigma_{\varepsilon}^{2}}$$

**References**

1. de Los Campos G, Gianola D, Rosa GJ, Weigel KA, Crossa J (2010) Semi-parametric genomic-enabled prediction of genetic values using reproducing kernel Hilbert spaces methods. Genet Res 92: 295–308.

2. Zhou X, Stephens M (2012) Genome-wide efficient mixed-model analysis for association studies. Nat Genet 44: 821–824.

3. Janss L, de los Campos G, Sheehan N, Sorensen D (2012) Inferences from Genomic Models in Stratified Populations. Genetics 192: 693–704. doi:10.1534/genetics.112.141143. PMID:22813891 PMCID:PMC3454890.
