# Supplementary material for: Genomic Heritability: What Is It?
Source: PLoS Genet. 2015 May 5;11(5):e1005048. doi: 10.1371/journal.pgen.1005048 (PMC4420472; doi:10.1371/journal.pgen.1005048)
Supplement: S4 Text — (DOCX) [file pgen.1005048.s004.docx]

## Supplementary Methods IV:

**Simulation of genotypes with simplified covariance structures**

We designed a simulation to generate genomes made of independent LD blocks. To this end (a) generated haplotypes according to a Markov process (see below), (b) randomly mated haplotypes to generate genotype blocks, and (c) randomly merged genotype blocks to generate a diploid genome. Genomes had 50,000 loci. In one of the simulation scenarios (SB=short blocks) each LD block had 5 loci and there were 10,000 blocks; in the other simulation scenario (LB=long blocks) each block had 50 loci and there were 1,000 blocks.

Markov Process Used to Sample one haplotypes (h):

1. Sample a probability (allele frequency, $\pi$) from a beta distribution $\pi\sim Beta(\alpha_{1_{\pi}},\alpha_{2_{\pi}})$
2. Sample a transition probability, $\theta$, from a beta distribution $\theta\sim Beta(\alpha_{1_{\theta}},\alpha_{2_{\theta}})$,
3. Sample an allele dummy variable, $d_{1}$, from Bernoulli distribution $d_{1}\sim Bernoulli(\pi)$
4. For *j* from *j=2 t*o *j=5* (SB scenario) or *j=50* (LB scenario) repeat the following tasks
   1. Sample a transition indicator variable, $t_{\left( j-1 \right),j}$, from Bernoulli distribution $t_{\left( j-1 \right),j}\sim Bernoulli( \theta)$
   2. If $t_{\left( j-1 \right),j}=0$ set $d_{j}=d_{j-1}$; otherwise sample $d_{j}\sim Bernoulli(\pi)$
5. Set the haplotypes genotype to be $h=\{ d_{1}, d_{2},\ldots d_{nL}\}$ where nL=5 (SB) or nL=50 (LB).

The parameters of the beta distribution from where allele frequencies were sampled were set to $\alpha_{1_{\pi}}=2$ and $\alpha_{2_{\pi}}=8$; this gives an average (median) allele frequency of 0.2 (0.180) and 0.025 (0.975) percentile of 0.028 (0.482) minor allele frequency. The transition probabilities, $\theta$, were also samples from a beta distribution with $\alpha_{1_{\theta}}=2$ and $\alpha_{2_{\theta}}=8$. In the fixed transition probability scenario (FTP) $\theta$ was sampled once for the entire genome and it was kept fixed at that value for the entire replicate. In random transition probabilities (RTP) scenarios, $\theta$ was sampled independently for each of the LD blocks. This generates a genome with LD patterns that are heterogeneous across blocks.

Genetic Values and Phenotypes

Out of the 50,000 loci 200 were randomly sampled to be QTL. In the SB scenarios, 200 blocks were randomly selected out of the 10,000, and a QTL was assigned to a randomly chosen locus within the LD block. In LB scenarios the QTL positions were assigned completely at random within the 50,000-loci of the genome. QTL effects and model residuals were sampled from normal densities with variance parameters chosen to target a trait heritability of 0.5. Phenotypes were constructed based on an additive model, as described in expression (1).

Analysis

For each MC replicate a total of 10,000 genotypes were generated; these constitute the conceptual ‘population’. These genotypes were used to compute the marker and QTL co-variance matrices needed, together with the QTL effects, needed to calculate the trait heritability and genomic heritability. One thousand individuals were randomly chosen from the simulated population. These were used to estimate genomic variances according to the maximum likelihood method described in section S2. For each replicate (a total of 3,000 MC replicates were used) and simulation scenario (SB-FTP, SB-RTP, LB-FTP, LB-RTP) estimates were derived using: only QTL genotypes (QTL), QTL plus the markers in LD with QTL (i.e., those in blocks harboring a QTL, this analysis was denoted as QTL+MRK.LD), all loci (ALL), only markers in LD with QTL (MRK.LD), all loci except the QTL loci (MRK.LD+MRK.LE) and only markers in LE with QTL (MR.LE).
