# Supplementary figures and images for: Genomic Heritability: What Is It?
Source: PLoS Genet. 2015 May 5;11(5):e1005048. doi: 10.1371/journal.pgen.1005048 (PMC4420472; doi:10.1371/journal.pgen.1005048)

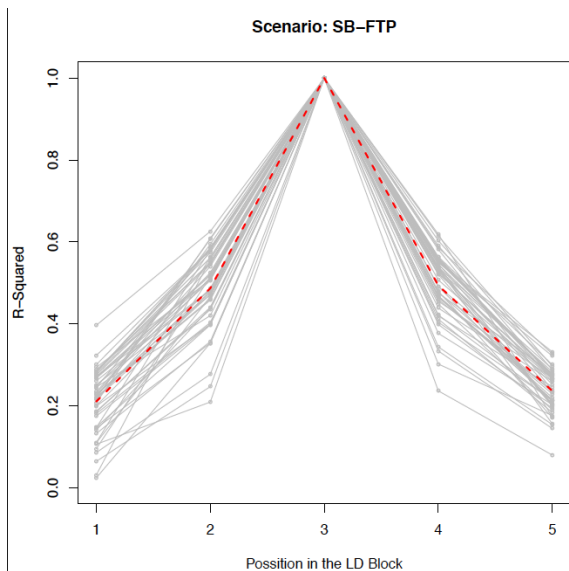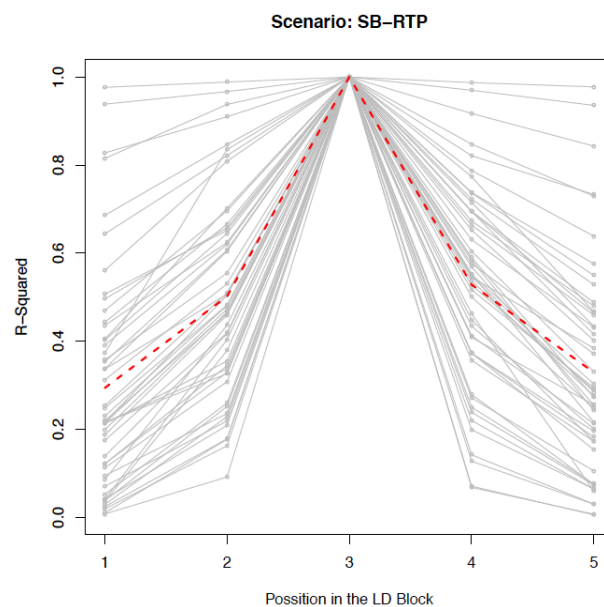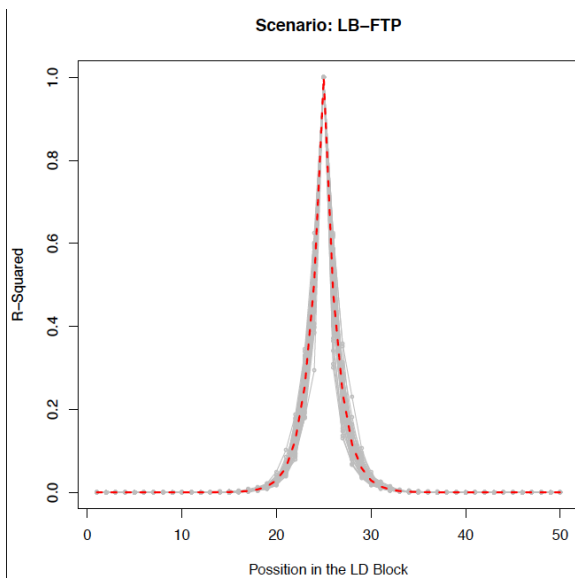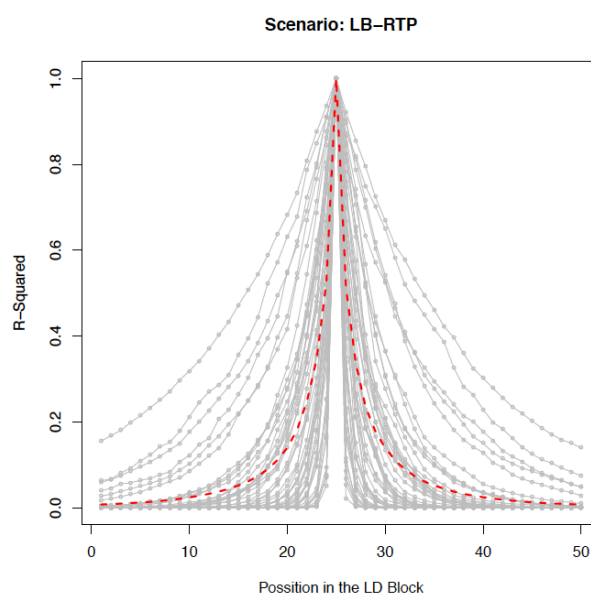

Supplement: S1 Fig — The grey lines give the realized LD patterns for different blocks and the red dashed line gives the average (across block) LD pattern. (PDF) [file pgen.1005048.s005.pdf]
